# Design and Implementation of an MPSoC-Based IMU/GNSS/Polarization Integrated Navigation System

**DOI:** 10.3390/s26196077

**Published:** 2026-09-25

**Authors:** Jie Wang, Pengwei Shen, Yuan Li

**Affiliations:** School of Information and Communication Engineering, North University of China, Taiyuan 030051, China; s202405004@st.nuc.edu.cn (J.W.); s202405040@st.nuc.edu.cn (P.S.)

**Keywords:** MPSoC, integrated navigation, polarized light, ESKF

## Abstract

To address the insufficient heading constraint of integrated navigation systems under GNSS-denied conditions and the high computational burden of high-resolution polarization-image processing, an IMU/GNSS/sky-polarization integrated navigation system based on a Zynq UltraScale+ MPSoC is designed and implemented. A PS–PL collaborative architecture is adopted, in which pixel-level polarization-image processing is accelerated in the programmable logic (PL). In contrast, frame-level polarization feature extraction, polarization-based heading estimation, and ESKF-based multi-source fusion are performed in the processing system (PS). The experimental results show that the average difference in AoP calculation between the IP core and the PC-based implementation is approximately $0.3^{\circ }$. In four repeated experiments conducted under clear-sky conditions, the position-deviation MAE remains below 0.34 m, while the heading deviation is generally around $2^{\circ }$. Three additional repeated experiments under cloudy conditions show that the position and heading deviations increase in some cases as the quality of the polarization observations decreases; nevertheless, the system maintains continuous and stable navigation solutions. Under a complete 120 s GNSS outage, the introduction of polarization-based heading observations reduces the heading MAE and RMSE by approximately 18% and 26%, respectively, demonstrating that polarization observations can effectively constrain inertial heading drift during prolonged GNSS outages.

## 1. Introduction

Global Navigation Satellite Systems (GNSSs) can provide long-term stable positioning and timing information; however, their performance is susceptible to building blockage, multipath effects, radio-frequency interference, and signal outages. Inertial navigation systems (INSs) offer the advantages of autonomy, continuity, and high update rates, but their position and attitude errors accumulate over time due to sensor biases and random noise. Therefore, integrated GNSS/INS navigation has been widely applied to vehicles and other mobile platforms. Nevertheless, when GNSS signals are unavailable for an extended period or are completely lost, additional external observations, independent of satellite signals, are still required to constrain inertial navigation errors [1].

Sky polarization offers a viable approach to autonomous directional sensing in GNSS-denied environments. Inspired by the orientation behavior of insects such as desert ants, which exploit celestial polarization patterns for navigation, Lambrinos et al. introduced a polarization compass for mobile robot navigation at an early stage [2], and this was followed by the gradual development of bio-inspired polarization navigation techniques [3,4]. Dupeyroux et al. utilized sky polarization information for bio-inspired heading estimation [5]. Wang et al. incorporated a polarization compass into a visual–inertial navigation system to provide an external constraint on inertial heading [6]. Zhao et al. investigated a multi-rate fusion method for integrating polarization-compass measurements with inertial navigation data [7]. Shen et al. further integrated MEMS-INS, GPS, and a polarization compass to improve the stability of multi-source navigation systems [8]. These studies demonstrate that polarization-based heading relies on a naturally available information source, requires no radio-frequency signals, and is not subject to the long-term error accumulation associated with inertial integration. It can therefore serve as an auxiliary directional observation under GNSS-restricted conditions.

However, real-world sky polarization patterns are affected by clouds, aerosols, local occlusions, and variations in the solar position. Hegedüs et al. investigated sky polarization patterns under thick cloud cover and demonstrated that clouds can alter the sky’s polarization characteristics [9]. Zhao et al. analyzed measured angle-of-polarization (AoP) distributions under different sky conditions to investigate the stability characteristics of sky polarization patterns and their navigation application [10]. To address abnormal polarization observations caused by complex environments, Dou et al. further employed an adaptive disturbance-rejection method to improve the reliability of polarization-aided integrated navigation [11]. Therefore, in practical integrated navigation systems, obtaining a polarization-derived heading alone is insufficient to guarantee satisfactory fusion performance. The reliability of polarization observations must also be evaluated based on polarization quality and the platform’s motion state.

Meanwhile, the development of division-of-focal-plane (DoFP) polarization imaging technology has enabled the simultaneous acquisition of polarization information at multiple orientations within a single image frame. Tyo et al. systematically reviewed the principles and representative architectures of imaging polarimetry [12]. Gruev et al. integrated micro-polarizer arrays with imaging sensors, enabling simultaneous focal-plane acquisition of polarization images [13]. Powell and Gruev further investigated calibration methods for DoFP polarimeters [14]. Compared with point-based polarization sensors, imaging-based approaches can provide substantially richer spatial polarization information. However, high-resolution polarization images require pixel-wise computation of numerous Stokes parameters, the degree of linear polarization (DoLP), and the AoP, which imposes a considerable computational burden for real-time processing [15]. Field-programmable gate arrays (FPGAs) offer advantages in parallel, pipelined, and streaming data processing. At the same time, high-level synthesis (HLS) enables high-level algorithms to be mapped onto hardware architectures through techniques such as pipelining and dataflow optimization [16,17]. System-on-chip FPGAs (SoC-FPGAs) integrate general-purpose processors and programmable logic on a single chip, providing a hardware foundation for the coordinated execution of navigation data processing and computationally intensive algorithms [18].

The above studies indicate that substantial progress has been made in polarization sensing, polarization-based heading estimation, and polarization/inertial integrated navigation. Nevertheless, further investigation is still required into heterogeneous integrated implementations that combine real-time processing of high-resolution polarization images with multi-source navigation fusion. To address this issue, this study designs and implements an IMU/GNSS/sky-polarization integrated navigation system based on a Zynq UltraScale+ MPSoC. A processing-system/programmable-logic (PS–PL) collaborative architecture is adopted, in which pixel-level acceleration of polarization-image processing is implemented in the PL. At the same time, frame-level polarization feature statistics, polarization-based heading estimation, and multi-source fusion based on an error-state extended Kalman filter (ESKF) are performed in the PS. The proposed system is validated through hardware–software computational consistency tests, repeated outdoor trajectory experiments, and complete GNSS-outage experiments.

## 2. Materials and Methods

### 2.1. System Architecture

A multi-source integrated navigation system consisting of a GNSS/IMU module, a division-of-focal-plane (DoFP) polarization camera, a Zynq UltraScale+ MPSoC processing platform, and a data storage unit was developed in this study. The overall system architecture is illustrated in Figure 1. The MPSoC consists of a processing system (PS) and programmable logic (PL). The PS runs an embedded Linux operating system and is primarily responsible for sensor data acquisition, related data processing, ESKF-based multi-source fusion, and data storage. The PL is primarily responsible for pixel-level accelerated processing of polarization images, including micro-polarizer macro-pixel extraction, computation of degree of linear polarization (DoLP) and angle of polarization (AoP), and preliminary screening of valid pixels.

After the corresponding sensors acquire data, the acquired data are processed through two data paths. The first path handles the IMU/GNSS data. The IMU/GNSS module transmits the IMU and GNSS measurements to the PS through a serial interface. The data are decoded by the IMU/GNSS Reception and Parsing module and then written into the DDR memory before being used in the subsequent ESKF-based multi-source fusion.

The second path handles the polarization-image data. The polarization camera transmits raw images to the PS through a USB 3.0 interface. The image data are first buffered in DDR memory and are subsequently transferred, under PS control, to the polarization-image-processing IP core in the PL through AXI DMA. After the IP core completes polarization data processing, the resulting data are transferred back to the DDR memory on the PS side via AXI DMA, thereby providing polarization observations for subsequent navigation fusion.

### 2.2. Coordinate Systems and Sensor Installation Relationships

To ensure a consistent coordinate representation for the IMU, GNSS, and polarization observations, the East–North–Up (ENU) coordinate system is adopted as the navigation frame n. The $x_{n}$, $y_{n}$ and $z_{n}$ axes point toward the east, north, and upward directions, respectively. The body b frame is defined according to the Right–Forward–Up (RFU) convention, with the IMU center taken as the origin. Accordingly, the $x_{b}$, $y_{b}$ and $z_{b}$ axes point toward the right, forward, and upward directions of the vehicle, respectively.

In this study, the heading angle ψ is defined as the angle between the forward body axis $y_{b}$ and the true north direction in the horizontal plane. True north is defined as 0°, and the heading increases clockwise within the range $\left[0^{\circ },360^{\circ }\right)$. This definition is used as the unified heading convention for the GNSS-derived course, polarization-based heading, and attitude output of the ESKF.

The polarization camera is rigidly mounted on the vehicle, with its optical axis approximately aligned with the $+z_{b}$ direction and pointing toward the sky. A fixed installation lever arm exists between the GNSS main antenna and the IMU center. This lever arm was determined through repeated mechanical measurements after installation and is expressed in the body frame as $l_{A}^{b}=[0,\;0.525,\;0{]}^{T}\;m,$ with an estimated measurement uncertainty of ±5 mm. The lever arm is compensated for in the subsequent GNSS position observation model. The fixed angular offset introduced by the polarization-camera installation and the coordinate transformation involved in polarization-based heading estimation is corrected using a fixed angular compensation parameter. These coordinate-system definitions and sensor installation relationships provide a unified reference for the subsequent polarization-based heading estimation and multi-source observation fusion.

### 2.3. Principle of Polarization Navigation and Polarization-Based Heading Estimation

After sunlight enters the atmosphere, it is scattered by air molecules and aerosols, forming a sky polarization pattern with a certain spatial distribution. Under the ideal single-scattering Rayleigh model, the polarization direction of the scattered light is perpendicular to the scattering plane defined by the solar ray and the observation direction. Therefore, directional information related to the solar azimuth can be obtained from the sky polarization pattern.

In this study, a division-of-focal-plane polarization camera is employed to acquire sky polarization information. For a single micro-polarizer macro-pixel, let the measured intensities at polarization orientations of 0°, 45°, 90°, and 135° be $I_{0}$, $I_{45}$, $I_{90}$, and $I_{135}$, respectively. The linear-polarization Stokes parameters are then expressed as [19]:

(1) $$S_{0}=\frac{1}{2}(I_{0}+I_{45}+I_{90}+I_{135})$$

(2) $$S_{1}=I_{0}-I_{90}$$

(3) $$S_{2}=I_{45}-I_{135}$$

where $S_{0}$ denotes the total intensity and reflects the local background irradiance, while $S_{1}$ and $S_{2}$ describe the intensity differences between two pairs of orthogonal linear-polarization components. Since the polarization camera used in this study mainly acquires linear-polarization information, the degree of linear polarization (DoLP) is adopted to characterize the strength of polarization:

(4) $$\mathrm{DoLP}=\frac{\sqrt{S_{1}^{2}+S_{2}^{2}}}{S_{0}}$$

The angle of polarization (AoP) describes the orientation of the linear-polarization direction relative to the image reference axis. To retain the quadrant information contained in $S_{1}$ and $S_{2}$, it is calculated using the two-argument arctangent function:

(5) $$\mathrm{AOP}=\frac{1}{2}\mathrm{atan}2\left(S_{2},S_{1}\right)$$

and is normalized to the range $\left[0^{\circ },180^{\circ }\right)$. Since the AoP of an individual pixel can be easily affected by clouds, buildings, ground reflections, and local noise, the heading is not directly estimated from single-pixel AoP values. Instead, polarization information within the valid-sky region is used to obtain a frame-level equivalent polarization angle $\bar{\theta }$. The detailed frame-level statistical procedure implemented on the PS side is described in Section 2.4.

Based on the observation position and time, the solar azimuth $A_{sun}$ is calculated [20]. Considering the coordinate-system definition given in Section 2.2, the camera mounting orientation, and the calibration offset of the micro-polarizer array, the frame-level polarization angle is converted into the polarization-heading axis as follows:

(6) $$\psi_{p,\mathrm{axis}}={\mathrm{wrap}}_{360}\left(A_{\mathrm{sun}}-\bar{\theta }-90^{\circ }+b_{p}\right)$$

where $b_{p}$ denotes the fixed angular compensation parameter in the polarization-based heading estimation chain. Prior to the formal experiments, four additional calibration datasets were collected, of which two were used for parameter estimation and the other two were reserved for independent validation. During calibration, a reliable GNSS-derived course was used as the engineering reference. Polarization-heading residuals were calculated for observations satisfying the prescribed motion-state and polarization-quality criteria, and robust statistics based on the median and median absolute deviation (MAD) were applied. Let the median residuals obtained from the two calibration datasets be ${\bar{r}}_{p,1}$ and ${\bar{r}}_{p,2}$, respectively. The compensation parameter is then corrected as follows:

(7) $$b_{p}=b_{p,0}-\mathrm{median}({\bar{r}}_{p,1},{\bar{r}}_{p,2})$$

With $b_{p,0}=-6.466^{\circ }$ and a calibration residual of $-11.453^{\circ }$, the final compensation parameter is obtained as follows: $b_{p}=4.987^{\circ }$. For the other two independent validation datasets that were not involved in parameter estimation, the signed median of the calibrated polarization-heading residuals is $3.094^{\circ }$, while the MAE and RMSE are $4.520^{\circ }$ and $5.972^{\circ }$, respectively. After calibration, $b_{p}$ is kept fixed throughout all subsequent formal experiments and polarization-based heading calculations.

Here, ${\mathrm{wrap}}_{360^{\circ }}(\cdot )$ denotes an angular wrapping operator that maps an arbitrary angle to $\left[0^{\circ },360^{\circ }\right)$. Similarly, ${\mathrm{wrap}}_{180^{\circ }}(\cdot )$ maps an angle to $\left[-180^{\circ },180^{\circ }\right)$ and is used to calculate the minimum circular angular difference between two headings.

Because a linear-polarization direction has no inherent arrow orientation and the AoP exhibits a $180^{\circ }$ periodicity, two candidate polarization headings exist [21]:

(8) $$\psi_{p,1}=\psi_{p,\mathrm{axis}}$$

(9) $$\psi_{p,2}={\mathrm{wrap}}_{360}(\psi_{p,\mathrm{axis}}+180^{\circ })$$

To determine the correct polarization-heading branch, the system first uses a reliable GNSS-derived course as the directional reference and selects the candidate with the smaller circular angular difference from the reference direction. Let the reference heading be $\psi_{\mathrm{ref}}$. The angular difference between each candidate heading and the reference direction is defined as

(10) $$d_{i}=|{\mathrm{wrap}}_{180}(\psi_{p,i}-\psi_{\mathrm{ref}})|,i=1,2$$

and the candidate corresponding to the smaller $d_{i}$ is selected as the current polarization heading $\psi_{p}$.

When the polarization-heading branch is initialized for the first time, a GNSS-derived course satisfying the required motion-state and quality criteria must be available for the initial directional disambiguation. Once the polarization branch has been established, if the GNSS-derived course becomes temporarily unavailable, branch continuity is maintained using the previously valid polarization-based heading together with the ESKF-predicted heading change between adjacent epochs, thereby reducing the likelihood of erroneous branch switching caused by the $180^{\circ }$ ambiguity. The continuity of this branch-maintenance strategy in the experimental data is further analyzed in Section 3.4.

The resulting $\psi_{p}$ is used as the polarization-based heading observation. Before being incorporated into the ESKF, its reliability is further evaluated using the DoLP, the proportion of valid-sky pixels, the vehicle motion state, and heading consistency, and the corresponding observation noise is adjusted based on the quality of the polarization observation. In practical environments, sky-region occlusion, reflections, and errors in solar-meridian extraction may reduce the reliability of polarization-based heading. Recent studies have therefore improved the robustness of polarization orientation estimation in complex environments through image registration and solar-meridian detection [22].

### 2.4. Design of the Polarization-Image-Processing IP Core

Based on the PS–PL functional partitioning described in Section 2.1, this section further presents the hardware implementation of the polarization-image-processing IP core and the frame-level polarization feature-extraction method. Considering the considerable pixel-wise computational load associated with 2448×2048 high-resolution polarization images, Vitis HLS is employed to implement the highly regular and stream-oriented polarization-parameter calculations in the PL. Pipelining and HLS Dataflow are further adopted to improve the parallelism of data processing.

According to the arrangement of the micro-polarizer array in the division-of-focal-plane polarization sensor, line buffers are used in the IP core to store adjacent pixels, and each macro-pixel is treated as the basic processing unit for extracting the four polarization-direction intensities. After macro-pixel reorganization, each original image frame produces 1224×1024 sets of polarization calculation results. A pipelined architecture is adopted for pixel input, and the initiation interval of the macro-pixel extraction loop is set to II=1, allowing the raw image data to be continuously streamed into the subsequent processing modules.

For the nonlinear operations involved in polarization-parameter calculation, a combination of fixed-point quantization and lookup-table-based approximation is adopted in the hardware implementation. Division is performed using a precomputed reciprocal ROM, while the inverse tangent operation is approximated using a lookup table. In addition, the DSP resources available in the PL are used to perform operations such as squaring and multiplication, thereby reducing the logic resource overhead associated with complex floating-point computation. An HLS Dataflow architecture connects the macro-pixel extraction and polarization-parameter calculation modules, enabling continuous, streaming execution of data reading, computation, and output.

The IP core’s input uses an 8-bit AXI4-Stream interface, while the output uses a 32-bit AXI4-Stream interface. The output corresponding to each macro-pixel consists of four 8-bit quantities, namely the average intensity, DoLP, AoP, and a validity flag, expressed as

(11) $$D_{\mathrm{out}}=\{I_{\mathrm{avg}},D_{\mathrm{DoLP}},D_{\mathrm{AoP}},M_{\mathrm{valid}}\}$$

where the AoP is represented by an 8-bit quantized value ranging from 0 to 255, corresponding to a polarization orientation within $\left[0^{\circ },180^{\circ }\right)$. The validity flag is generated according to the current DoLP threshold. This threshold is dynamically written to the IP core via the AXI4-Lite interface by the PS.

After the pixel-level polarization results generated in the PL are transferred back to the DDR memory on the PS side through AXI DMA, frame-level polarization feature statistics are further performed by the PS. First, underexposed and overexposed regions are removed according to the average macro-pixel intensity. Only pixels satisfying $30\leq I_{\mathrm{avg}}\leq 240$ are retained. A new DoLP screening threshold is then calculated from the DoLP histogram of the current frame using Otsu’s method. To reduce threshold fluctuations between adjacent image frames, the threshold is updated using the following smoothing strategy:

(12) $$T_{k}=0.8T_{k-1}+0.2T_{\mathrm{Otsu},k}$$

For the valid pixels that remain after the final screening process, the AoP values cannot be directly averaged arithmetically because of their periodicity. Therefore, the PS employs double-angle vector statistics to obtain the frame-level equivalent polarization angle required in Section 2.3. Let the set of valid pixels be denoted by Ω. The accumulated cosine and sine components are calculated as follows:

(13) $$C=\sum_{i\in \Omega }\cos(2\theta_{i})$$

(14) $$S=\sum_{i\in \Omega }\sin(2\theta_{i})$$

and the corresponding frame-level equivalent polarization angle is given by

(15) $$\bar{\theta }=\frac{1}{2}\mathrm{atan}2(S,C)$$

where i denotes the index of a valid pixel, $\theta_{i}$ is the AoP of the i-th valid pixel, C and S represent the accumulated cosine and sine components of the double-angle vectors, respectively. $\bar{\theta }$ denotes the equivalent polarization angle of the current image frame. The double-angle transformation maps linear-polarization orientations separated by $180^{\circ }$ onto the same circular direction, thereby avoiding abrupt angular changes that may occur when AoP values span $0^{\circ }$ to $180^{\circ }$ and are directly averaged.

The polarization-image-processing IP core generated through HLS is shown in Figure 2. The video_in input interface has an 8-bit data width, whereas the video_out output interface has a 32-bit data width. Threshold configuration and runtime control are implemented through the AXI4-Lite interface.

The main resource utilization and timing results of the polarization-image-processing IP core obtained after HLS are summarized in Table 1.

It should be noted that the timing results obtained from HLS primarily reflect the theoretical implementation capability of the computational core itself, whereas the actual processing performance of the MPSoC system is also affected by DDR access, AXI DMA data transfer, PS–PL data movement, and frame-level statistical processing on the PS. Therefore, additional on-board processing-performance tests were conducted on the actual MPSoC platform, and the corresponding results are presented in Section 3.2.

### 2.5. ESKF-Based Multi-Source Integrated Navigation Fusion

To achieve unified fusion of the IMU, GNSS, and polarization-based heading measurements, an Error-State Extended Kalman Filter (ESKF) is employed for navigation-state estimation.

The IMU is used for continuous state propagation, while the GNSS provides position observations and heading constraints when sufficient vehicle motion is available. The polarization-based heading obtained in Section 2.3 serves as an additional external directional observation for attitude correction. The ESKF estimates the small error state between the nominal and true states in a linearized error-state filter and injects the estimated error into the nominal state after each measurement update, thereby keeping the linearization error within bounds.

For notational simplicity, define $C\equiv C_{b}^{n}$, where C denotes the attitude rotation matrix from the body frame b to the navigation frame n.

The nominal state consists of position, velocity, attitude, and IMU biases, and is expressed as

(16) $$X=\left\{p^{n},v^{n},C,b_{g},b_{a}\right\}$$

The corresponding error state is defined as

(17) $$\delta x={\left[\begin{array}{ccccc} \delta p^{T} & \delta v^{T} & \delta \theta^{T} & \delta b_{g}^{T} & \delta b_{a}^{T} \end{array}\right]}^{T}\in R^{15}$$

where $p^{n}$ and $v^{n}$ denote the position and velocity of the IMU center in the navigation frame, respectively; $b_{g}$ and $b_{a}$ denote the gyroscope and accelerometer biases, respectively; δp, δv, and δθ represent the position, velocity, and small-angle attitude errors; and δbg and δba are the corresponding bias errors. All of these error quantities are three-dimensional vectors.

#### 2.5.1. IMU State Prediction

During each inertial propagation interval, the IMU measurements are first corrected using the current bias estimates:

(18) $${\tilde{\omega }}_{k-1}^{b}=\omega_{k-1}^{b}-{\hat{b}}_{g}$$

(19) $${\tilde{f}}_{k-1}^{b}=f_{k-1}^{b}-{\hat{b}}_{a}$$

where $\omega_{k-1}^{b}$ and $f_{k-1}^{b}$ are the angular velocity and specific force measured by the IMU, respectively, while ${\tilde{\omega }}_{k-1}^{b}$ and ${\tilde{f}}_{k-1}^{b}$ denote the corresponding bias-corrected measurements.

For a sampling interval Δt, the nominal attitude is propagated using an incremental rotation:

(20) $${\hat{C}}_{k|k-1}={\hat{C}}_{k-1|k-1}\mathrm{Exp}\left({\left[{\tilde{\omega }}_{k-1}^{b}\Delta t\right]}_{\times }\right)$$

The velocity and position are propagated as follows:

(21) $${\hat{v}}_{k|k-1}^{n}={\hat{v}}_{k-1|k-1}^{n}+\left({\hat{C}}_{k-1|k-1}{\tilde{f}}_{k-1}^{b}+g^{n}\right)\Delta t$$

(22) $${\hat{p}}_{k|k-1}^{n}={\hat{p}}_{k-1|k-1}^{n}+{\hat{v}}_{k-1|k-1}^{n}\Delta t+\frac{1}{2}\left({\hat{C}}_{k-1|k-1}{\tilde{f}}_{k-1}^{b}+g^{n}\right)\Delta t^{2}$$

where $g^{n}$ is the gravity vector expressed in the navigation frame. The operator ${\left[\cdot \right]}_{\times }$ denotes the skew-symmetric matrix constructed from a three-dimensional vector, and Exp(⋅) denotes the exponential mapping from a rotation vector to a rotation matrix.

Meanwhile, the covariance of the corresponding error state is propagated using the linearized error model:

(23) $$P_{k|k-1}=\Phi_{k-1}P_{k-1|k-1}\Phi_{k-1}^{T}+Q_{k-1}$$

where $\Phi_{k-1}$ is the discrete state-transition matrix and $Q_{k-1}$ is the discrete process-noise covariance matrix, which accounts for the effects of gyroscope noise, accelerometer noise, and random walks of the two sensor biases.

#### 2.5.2. GNSS Position Measurement Update

As described in Section 2.2, a fixed lever arm $l_{A}^{b}$ exists between the GNSS position-reference point and the IMU center. Therefore, the GNSS position observation model is expressed as

(24) $$z_{k}^{g}=p_{k}^{n}+C_{k}l_{A}^{b}+n_{k}^{g}$$

and the corresponding position innovation is

(25) $$\rho_{k}^{g}=z_{k}^{g}-\left({\hat{p}}_{k|k-1}^{n}+{\hat{C}}_{k|k-1}l_{A}^{b}\right)$$

After considering the attitude error induced by the lever arm during vehicle motion, the corresponding linearized observation matrix can be written as

(26) $$H_{k}^{\mathrm{pos}}=\left[I_{3\times 3}\;\;\;0_{3\times 3}\;\;\;-{\hat{C}}_{k|k-1}{\left[l_{A}^{b}\right]}_{\times }\;\;\;0_{3\times 3}\;\;\;0_{3\times 3}\right]$$

where $n_{k}^{g}$ denotes the corresponding measurement noise.

The covariance of the GNSS position observation is adjusted based on the current positioning status and quality indicators, such as HDOP. If the measurement innovation fails the consistency test, the corresponding GNSS position update is rejected. In practical operation, the vehicle’s motion state and heading reliability are also considered to limit direct attitude corrections under low-dynamic conditions or when the motion direction is unreliable.

#### 2.5.3. GNSS Course Update

Unlike directly using a dual-antenna heading solution, the fusion program in this study derives the vehicle course from consecutive GNSS position changes. Let the horizontal displacement between two GNSS positions satisfying the specified time and displacement conditions be ΔE  and ΔN. The vehicle course is then calculated as follows:

(27) $$\psi_{k}^{g}={\mathrm{wrap}}_{360^{{}^{\circ}}}\left[\mathrm{atan}2\left(\Delta E_{k},\Delta N_{k}\right)\right]$$

The calculated $\psi_{k}^{g}$ is regarded as a valid GNSS-derived course only when the GNSS position is valid and the predefined requirements for minimum vehicle speed, minimum displacement, course freshness, and other conditions are satisfied. The relevant time intervals and threshold parameters are specified uniformly in the experimental parameter settings.

The corresponding heading innovation is

(28) $$r_{k}^{g}={\mathrm{wrap}}_{180^{{}^{\circ}}}\left(\psi_{k}^{g}-{\hat{\psi }}_{k|k-1}\right)$$

where ${\hat{\psi }}_{k|k-1}$ denotes the current ESKF-predicted heading.

In practice, the GNSS-derived course is primarily used to correct attitude errors. Through state correlations, a limited correction of the vertical-axis gyroscope bias is also permitted, thereby reducing heading drift during vehicle motion.

#### 2.5.4. Polarization-Based Heading Measurement Update

The polarization-based heading $\psi_{k}^{p}$ is obtained using the method described in Section 2.3. However, not every polarization observation is directly incorporated into the filter. The navigation system first performs quality screening based on the mean DoLP, the proportion of valid-sky pixels, vehicle speed, roll angle, pitch angle, and yaw rate [23]. When a valid GNSS-derived course is available, the consistency between the polarization-based heading and the GNSS-derived course is additionally checked.

After the above conditions are satisfied, the innovation of the polarization-based heading relative to the predicted heading is calculated as follows:

(29) $$r_{k}^{p}={\mathrm{wrap}}_{180^{{}^{\circ}}}\left(\psi_{k}^{p}-{\hat{\psi }}_{k|k-1}\right)$$

The polarization-based heading update is performed only when the innovation satisfies the prescribed threshold. In the data-acquisition program, different innovation thresholds are used depending on whether a valid GNSS-derived course is available, so as to prevent the polarization observation from imposing an excessively strong constraint after the GNSS directional reference is lost.

Considering that the reliability of polarization observations is closely related to the quality of sky polarization information, the standard deviation of the polarization-based heading observation is adaptively adjusted according to the mean DoLP ${\bar{D}}_{k}$ and the proportion of valid-sky pixels $\eta_{k}$:

(30) $${\tilde{\sigma }}_{k}^{p}=\frac{0.5}{\max\left(\eta_{k},0.01\right)\max\left({\bar{D}}_{k},0.01\right)}$$

and is further constrained as follows:

(31) $$\sigma_{k}^{p}=\min\left\{\sigma_{\max},\max\left(\sigma_{\min},{\tilde{\sigma }}_{k}^{p}\right)\right\}$$

Therefore, when the polarization quality is high, $\sigma_{k}^{p}$ decreases and the corresponding polarization observation receives a larger weight in the filter. Conversely, when the DoLP or the proportion of valid-sky pixels decreases, $\sigma_{k}^{p}$ increases, and the influence of the current polarization-heading update on the filter state decreases.

The polarization-based heading is treated as a scalar attitude observation, and its linearized measurement model is expressed as

(32) $$r_{k}^{p}=H_{k}^{\psi }\delta x_{k|k-1}+n_{k}^{p}$$

where the corresponding linearized observation matrix is

(33) $$H_{k}^{\psi }=\left[\begin{array}{ccccc} 0_{1\times 3} & 0_{1\times 3} & h_{k}^{\psi } & 0_{1\times 3} & 0_{1\times 3} \end{array}\right]$$

where $h_{k}^{\psi }$ denotes the local Jacobian of the heading angle with respect to the three-dimensional small-angle attitude error.

#### 2.5.5. ESKF Measurement Update

For GNSS position, GNSS-derived course, and polarization-based heading observations that pass the corresponding validity checks, a unified ESKF measurement-update formulation is adopted.

Let the dimension of the current observation be m. The innovation vector is then given by $r_{k}\in R^{m}$, and the corresponding observation matrix is $H_{k}\in R^{m\times 15}$. For GNSS position observations, m=3, whereas for both GNSS-derived course and polarization-based heading observations, m=1.

The innovation covariance matrix is calculated as follows:

(34) $$S_{k}=H_{k}P_{k|k-1}H_{k}^{T}+R_{k}$$

and the Kalman gain is

(35) $$K_{k}=P_{k|k-1}H_{k}^{T}S_{k}^{-1}$$

For the subsequent unified measurement update, the estimated error state is obtained as follows:

(36) $$\delta {\hat{x}}_{k|k}=K_{k}r_{k}$$

where $H_{k}$, $r_{k}$, and $R_{k}$ denote the observation matrix, innovation vector, and measurement-noise covariance corresponding to the current observation.

To maintain the numerical stability of the covariance matrix after the measurement update, the updated covariance is calculated using the Joseph form:

(37) $$P_{k|k}=\left(I_{15}-K_{k}H_{k}\right)P_{k|k-1}{\left(I_{15}-K_{k}H_{k}\right)}^{T}+K_{k}R_{k}K_{k}^{T}$$

After completion of the measurement update, the estimated error state is injected into the nominal state:

(38) $$\left\{\begin{array}{c} {\hat{p}}_{k|k}^{n}={\hat{p}}_{k|k-1}^{n}+\delta {\hat{p}}_{k|k}\\ {\hat{v}}_{k|k}^{n}={\hat{v}}_{k|k-1}^{n}+\delta {\hat{v}}_{k|k}\\ {\hat{C}}_{k|k}={\hat{C}}_{k|k-1}\exp\left({\left[\delta {\hat{\theta }}_{k|k}\right]}_{\times }\right)\\ {\hat{b}}_{g}\leftarrow {\hat{b}}_{g}+\delta {\hat{b}}_{g}\\ {\hat{b}}_{a}\leftarrow {\hat{b}}_{a}+\delta {\hat{b}}_{a} \end{array}\right.$$

After error injection, the error state is reset to zero and the filter proceeds to the next inertial propagation interval. The acquisition program performs measurement updates sequentially based on the validity of GNSS position, GNSS-derived course, and polarization-based heading observations. When any external observation is unavailable, only the corresponding measurement update is skipped, while continuous IMU-based state propagation remains unaffected. The overall ESKF procedure is illustrated in Figure 3.

## 3. Results

### 3.1. Experimental Platform and Setup

To conduct outdoor tests of the proposed integrated navigation system, an experimental platform consisting of an HI32 dual-antenna GNSS/IMU module, a MER2-502-79U3M POL polarization camera, a Xilinx ZYNQ MPSoC processing platform, and an external power supply was constructed. The experimental platform is shown in Figure 4. The main sensor specifications are summarized in Table 2.

The outdoor experiments were conducted at the western plaza of Building B in the Science Complex of the North University of China, Taiyuan, Shanxi Province (geographic coordinates: 112°26′36′′ E, 38°1′1′′ N). The experiments were conducted on 24 July and 12 September 2026, and a total of seven independent repeated runs, denoted as Run 1–Run 7, were completed. Run 1–Run 4 were conducted under clear-sky conditions, whereas Run 5–Run 7 were conducted under cloudy conditions. The same hardware configuration and fusion parameters were used in all runs, while sky polarization images, IMU measurements, and GNSS data were recorded synchronously. Since several thresholds are involved in the construction of the GNSS-derived course and the screening of polarization observations, the key parameters that have a relatively significant influence on the fusion results are summarized in Table 3 to improve the reproducibility of the algorithm implementation.

### 3.2. Validation and Performance Analysis of the Polarization-Image-Processing IP Core

To verify the computational consistency of the polarization-image-processing IP core implemented in the PL, the AoP results obtained using a double-precision floating-point algorithm on the PC were taken as the reference. The IP core then processed the same raw polarization images, and the results obtained using the two implementations were compared.

Figure 5 shows five representative time instants from one experimental dataset. The first row presents the original polarization images, while the second and third rows show the AoP distributions calculated by the PC and the IP core, respectively.

As shown in Figure 5, the dominant AoP regions, spatial distributions, and temporal variation trends obtained by the two implementations are generally consistent. No obvious regional discontinuities or directional anomalies are observed, indicating that the fixed-point hardware implementation preserves the principal polarization-distribution characteristics of the PC-based floating-point algorithm.

To further evaluate the numerical stability of the hardware implementation, the minimum wrapped angular difference in AoP between the IP core and the PC was calculated frame-by-frame for all seven experiments. The corresponding results are summarized in Table 4.

As shown in Table 4, the overall MAE between the AoP results obtained by the PC-based implementation and the IP core is approximately $0.3^{\circ }$, and the statistical results under different weather conditions remain at a similar level. Overall, the fixed-point quantization, lookup-table approximation, and hardware data path do not introduce any noticeable additional numerical bias as the sky conditions change. These results indicate that the proposed IP core can reliably reproduce the AoP calculation results of the PC-based floating-point algorithm and can meet the computational requirements of subsequent frame-level polarization feature extraction and heading estimation.

To further evaluate the processing performance on the actual MPSoC platform, on-board tests were conducted for both the PS-only and PS–PL implementations using the same 2448×2048 Mono8 raw images. In the PS-only implementation, both pixel-level polarization computation and frame-level statistical processing are performed on the PS. In the PS–PL implementation, the pixel-level computation is deployed in the PL, while the frame-level processing is performed on the PS after the processed results are transferred back from the PL. The measured results are summarized in Table 5.

The end-to-end processing time of the PS–PL implementation is reduced from 107.416 ms/frame to 97.163 ms/frame, while the sustained processing rate increases from 9.310 fps to 10.292 fps. The pixel-level computation kernel on the PS was further timed independently, with an average execution time of 90.818 ms/frame. In comparison, the DMA + PL stage requires approximately 50.173 ms/frame, corresponding to a speedup of approximately 1.81× for the main pixel-level processing stage.

Considering the differences in image resolution among previous studies, the raw input-pixel throughput is further calculated from the input resolution and the reported frame rate to provide a unified comparison metric. The proposed implementation is then compared with representative real-time polarization-image hardware implementations, as summarized in Table 6.

With an input resolution of 2448×2048, the proposed system achieves an actual end-to-end processing rate of 10.292 fps, corresponding to an input-pixel throughput of approximately 51.60 MPixel/s. The proposed IP core utilizes 2182 LUTs, 1263 FFs, 7 BRAMs, and 5 DSPs. For comparison, the DoFP processing pipeline reported by Lapray et al. [25] utilizes 9558 LUTs, 7328 registers, 4 BRAMs, and 12 DSPs.

Because the hardware platforms, processing pipelines, and timing boundaries differ among the compared studies, the above comparison is primarily intended to characterize the scale of hardware resource utilization and practical data-processing capability rather than to provide an absolute performance ranking across different hardware architectures. Combined with the measured on-board results, the proposed PS–PL architecture achieves a favorable engineering trade-off between high-resolution polarization-image-processing capability and hardware resource overhead.

### 3.3. Trajectory Solution and Repeatability Validation

To evaluate the continuous navigation capability and repeatability of the proposed integrated navigation system under actual motion conditions, the trajectory and heading results from seven independent outdoor experiments were compared. Since no independent high-precision navigation ground truth was available during the experiments, the preset motion route and the theoretical motion directions of the stable straight-line segments were used as engineering references. Therefore, the statistical results in this section are intended to evaluate the deviation of the system relative to the engineering references and the consistency among repeated experiments, rather than the absolute positioning and heading accuracy of the system.

Figure 6 shows the two-dimensional trajectory comparison results for the seven experiments. As can be seen from Figure 6, all trajectories generally reproduce the preset motion route well, and the trajectory shapes obtained from different runs are relatively similar. The more noticeable deviations mainly occur near turning points and trajectory endpoints, whereas no persistent deviation or obvious divergence is observed along the major straight-line segments. These results indicate that the system is capable of maintaining stable and continuous position estimation.

To quantitatively evaluate the consistency of the trajectory solutions, position deviations from the preset trajectory were calculated over the stable-motion intervals. The results are summarized in Table 7.

As shown in Table 7, the position-deviation MAE values for Run 1–Run 4 range from 0.277 to 0.336 m, and the results are relatively close to one another, indicating good repeatability under clear-sky conditions. For Run 5–Run 7, the position-deviation MAE values range from 0.618 to 1.475 m. Run 7 remains below 1 m, whereas larger position deviations are observed in Run 5 and Run 6. Overall, all seven experiments maintain motion trends consistent with the preset route, and no obvious position divergence is observed during continuous operation.

The heading deviations relative to the preset motion direction are summarized in Table 8.

As shown in Table 8, the heading-deviation MAE values for Run 1–Run 4 range from $1.557^{\circ }$ to $2.256^{\circ }$, with an average of $1.827^{\circ }$. The results are generally at a similar level, indicating good repeatability of the heading output under clear-sky conditions. For Run 5–Run 7, the heading-deviation MAE values are $3.909^{\circ }$, $1.803^{\circ }$, and $3.533^{\circ }$, respectively, with an average of $3.082^{\circ }$. Among them, Run 6 remains at a level comparable to Run 1–Run 4, whereas larger deviations are observed in Run 5 and Run 7, indicating that the effectiveness of polarization-based heading in constraining the integrated navigation state varies to some extent as the environmental conditions change.

It should be noted that Run 5–Run 7 were conducted on a different date to Run 1–Run 4 and were directly corrected using the engineering reference direction determined from the clear-sky experiments and kept fixed thereafter. Therefore, the corresponding results are affected by both the change in weather conditions and the differences between experimental batches, and the increase in deviation cannot be attributed entirely to cloudy weather.

To further analyze the variation in polarization-observation quality under different weather conditions, statistics were calculated for the valid polarization observations. Under clear-sky conditions, the median DoLP and valid-sky proportion are approximately 0.240 and 0.159, respectively. Under cloudy conditions, these values decrease to approximately 0.210 and 0.114, respectively. This indicates that both the strength of the sky polarization characteristics and the effective image regions that can be stably used for heading estimation decrease under cloudy conditions, thereby weakening the effective constraint provided by polarization-based heading on the filter state.

The system performs quality screening based on the DoLP, valid-sky proportion, vehicle attitude, and heading innovation, and adaptively adjusts the observation noise according to the quality of the polarization observations. When the polarization quality decreases, its weight in the ESKF update is correspondingly reduced; when the quality falls below the prescribed threshold, the corresponding polarization update is rejected. Therefore, although the heading deviation increases in some cases under cloudy conditions, all seven experiments still maintain continuous trajectory and heading estimation without obvious sustained divergence. These results indicate that the adopted quality-control and adaptive-fusion strategy can, to some extent, mitigate the influence of environmental changes on the navigation state.

### 3.4. Validation of Polarization-Aided Heading Under GNSS-Outage Conditions

To further evaluate the capability of polarization-based heading to constrain inertial heading drift when a GNSS is unavailable, complete GNSS-outage scenarios were constructed using the four actual road-test datasets from Run 1–Run 4 and evaluated through offline replay. The outdoor experiments described above comprised seven datasets in total, among which Run 5–Run 7 were mainly used to assess environmental adaptability under different weather conditions. Therefore, Run 1–Run 4, which were acquired under relatively consistent weather conditions, were selected for the GNSS-outage experiments in this section to reduce the additional influence of weather-induced variations in polarization-observation quality.

Before the beginning of each outage interval, all comparison schemes used the same GNSS/IMU fusion state. After entering the outage interval, both GNSS position updates and GNSS-derived course updates were disabled to ensure identical initial states and external constraints for the two outage schemes. The baseline scheme relied solely on IMU propagation and is therefore referred to as IMU-only. In the polarization-aided scheme, polarization-based heading observations satisfying the validity criteria described in Section 2.5 were incorporated on top of the same inertial propagation process. Therefore, the primary difference between the two schemes is whether polarization-based heading observations participate in the ESKF update.

The complete GNSS/IMU navigation solution was used only as an engineering reference for heading-deviation evaluation and was not involved in the state updates during the outage interval. Since polarization-based heading estimation requires the solar azimuth, the first valid latitude and longitude obtained at the beginning of each run were used as the fixed geographic position, while the solar azimuth was calculated using the continuous UTC timestamps. This prevents real-time GNSS position information from being continuously used within the GNSS-outage interval. Seven GNSS-outage durations were considered: 20, 30, 40, 50, 60, 90, and 120 s.

Figure 7 compares the heading deviations of the two schemes under a representative GNSS-outage condition. During the initial stage of the outage, the differences between the two schemes are relatively small because the inertial solution can still maintain a comparatively stable heading over a short period. As the outage duration increases, however, the IMU-only solution is increasingly affected by accumulated gyroscope errors. In contrast, the polarization-based heading provides an external directional constraint independent of inertial integration, thereby limiting the continued growth of heading error during part of the prolonged outage interval.

To further quantify the benefit of polarization assistance for different outage durations, the heading MAE and RMSE of each valid case were calculated relative to the complete GNSS/IMU engineering reference. The results are summarized in Table 9.

As shown in Table 9, the effectiveness of polarization assistance exhibits a clear dependence on the outage time scale. During the short- and medium-duration outage intervals of 20–60 s, the relative performance of the two schemes fluctuates. An improvement is already observed at 40 s, whereas no consistent advantage is obtained at 20, 30, 50, or 60 s. This indicates that when inertial heading drift is still relatively small, the dynamic variation in the polarization observations and the intermittent availability of valid polarization updates may offset part of the benefit provided by the external heading constraint.

To further investigate the causes of these performance fluctuations, the polarization-observation quality, vehicle attitude, heading continuity, and solar-azimuth calculation error were analyzed using the Run 1–Run 4 datasets. Among the 19,711 valid polarization frames, the overall median valid-sky proportion is 0.1605, with a 5th–95th percentile range of 0.1311–0.1705. The median DoLP is 0.2395. These results indicate that the quality of the polarization observations varies dynamically during vehicle motion and affects the update weight of the polarization-based heading in the ESKF through the adaptive observation-noise model.

Among the 2262 candidate polarization updates, 631 were rejected because the roll or pitch angle exceeded the prescribed thresholds, accounting for 27.9% of the total. This indicates that vehicle attitude disturbances are one of the main causes of intermittent polarization updates.

Meanwhile, the maximum circular change between two consecutive valid polarization-based heading observations is $13.049^{\circ }$, and no abnormal jump exceeding $90^{\circ }$ is observed. Therefore, no obvious erroneous branch switching caused by the $180^{\circ }$ ambiguity is observed under the experimental conditions considered in this study.

To assess the influence of using a fixed initial geographic position for solar-azimuth calculation during the outage interval, the solar azimuth was recalculated using the position at each epoch for comparison. Across the four experiments, the maximum horizontal displacement relative to the initial position is approximately 52 m. The MAE and RMSE of the solar-azimuth difference introduced by the fixed-position approximation are $0.000142^{\circ }$ and $0.000207^{\circ }$, respectively, with a maximum value of only $0.000479^{\circ }$. These errors are far smaller than the degree-level variation in the polarization-based heading itself. Therefore, neither erroneous $180^{\circ }$ branch switching nor the solar-azimuth error introduced by the fixed-position approximation is a major source of the short- and medium-duration performance fluctuations.

Overall, the performance fluctuations during short- and medium-duration GNSS outages are mainly associated with the still-limited accumulation of inertial heading drift, variations in polarization-observation quality, and intermittent updates caused by vehicle attitude disturbances. As the outage duration increases further, inertial heading drift gradually accumulates and the external directional constraint provided by polarization-based heading becomes more evident. Under the 90 s outage condition, polarization assistance reduces the heading MAE and RMSE by approximately 6% and 15%, respectively. Under the 120 s outage condition, the two metrics decrease from $2.415^{\circ }$ and $2.922^{\circ }$ to $1.982^{\circ }$ and $2.170^{\circ }$, corresponding to improvements of approximately 18% and 26%, respectively. These results indicate that the primary advantage of polarization information lies in continuously constraining inertial heading drift during prolonged GNSS unavailability rather than providing an immediate improvement under every short-duration outage condition.

## 4. Conclusions

In this study, an IMU/GNSS/sky-polarization integrated navigation system based on a Zynq UltraScale+ MPSoC was designed and implemented. Through PS–PL collaboration, polarization-image processing, polarization-based heading estimation, and multi-source navigation fusion were jointly realized. The experimental results demonstrate that the AoP results obtained by the polarization-image-processing IP core implemented in the PL are highly consistent with those calculated using the floating-point algorithm on the PC, with an average difference of approximately 0.3°. This indicates that the fixed-point quantization and lookup-table implementation can reliably preserve the AoP calculation results and meet the computational requirements of subsequent frame-level polarization feature extraction and heading estimation. On-board measurements further show that the PS–PL implementation achieves an end-to-end processing time of 97.163 ms/frame and a sustained processing rate of 10.292 fps, while providing an approximately 1.81× speedup in the main pixel-level processing stage compared with the PS-based software implementation.

Across the seven repeated trajectory experiments, the system maintains continuous trajectory and heading outputs. In the four clear-sky experiments, the position-deviation MAE remains below 0.34 m, while the heading deviation is generally around $2^{\circ }$, demonstrating good repeatability. In the three cloudy-weather experiments, the position and heading deviations increase in some cases; nevertheless, the overall trajectory structure and heading solution remain continuous, with no obvious sustained divergence. The observed changes in polarization quality under cloudy conditions indicate that clouds and local sky occlusions reduce the amount of effective polarization information, while the quality-screening and adaptive observation-weighting mechanisms can, to some extent, mitigate the influence of low-quality polarization observations on the fused navigation state. It should be emphasized that no independent high-precision navigation system was available as ground truth in these experiments. Therefore, the above results mainly reflect deviations relative to the preset route and motion direction, as well as consistency among repeated experiments, and should not be interpreted as measures of the absolute navigation accuracy of the system.

The experiments under GNSS-outage conditions further demonstrate that the effectiveness of polarization assistance depends on the outage duration. During short- and medium-duration outages, the inertial heading error has not yet accumulated significantly, while polarization measurements still contain a certain level of noise; therefore, the relative performance of the two schemes fluctuates under different operating conditions. As the outage duration increases, the role of polarization-based heading as an external directional constraint becomes more evident. Under a 90s GNSS outage, polarization assistance improves the heading MAE and RMSE by approximately 6% and 15%, respectively. When the outage duration is extended to 120s, the corresponding improvements increase to approximately 18% and 26%. These results indicate that the principal value of polarization information lies in constraining the continued accumulation of inertial heading drift during prolonged GNSS unavailability, rather than providing an immediate improvement during all short-term outage intervals.

In addition, complex weather conditions, multiple scattering, and local occlusions may affect the spatial distribution of sky polarization patterns, thereby reducing the reliability of polarization-based heading measurements. Improving the environmental adaptability of polarization observations therefore remains an important research direction in bio-inspired polarization navigation [26]. The experiments conducted in this study cover both clear-sky and generally cloudy conditions and include local sky occlusions in sections of the motion route adjacent to buildings. Nevertheless, the environmental and motion conditions considered remain limited. Systematic tests have not yet been conducted under thick cloud cover, fully overcast conditions, prolonged large-area sky occlusion, or a wide range of solar-elevation angles. In addition, the current experimental platform primarily operates under low-speed planar motion and does not sufficiently cover highly dynamic conditions such as sharp turns, pronounced speed variations, or large roll and pitch disturbances. Future work will therefore focus on long-duration experiments under more complex weather conditions, persistent occlusions, and highly dynamic motion, while introducing an independent high-precision navigation reference to further evaluate the absolute positioning and heading performance of the system and the environmental robustness of the polarization-aided navigation method.

## Figures and Tables

**Figure 1:**
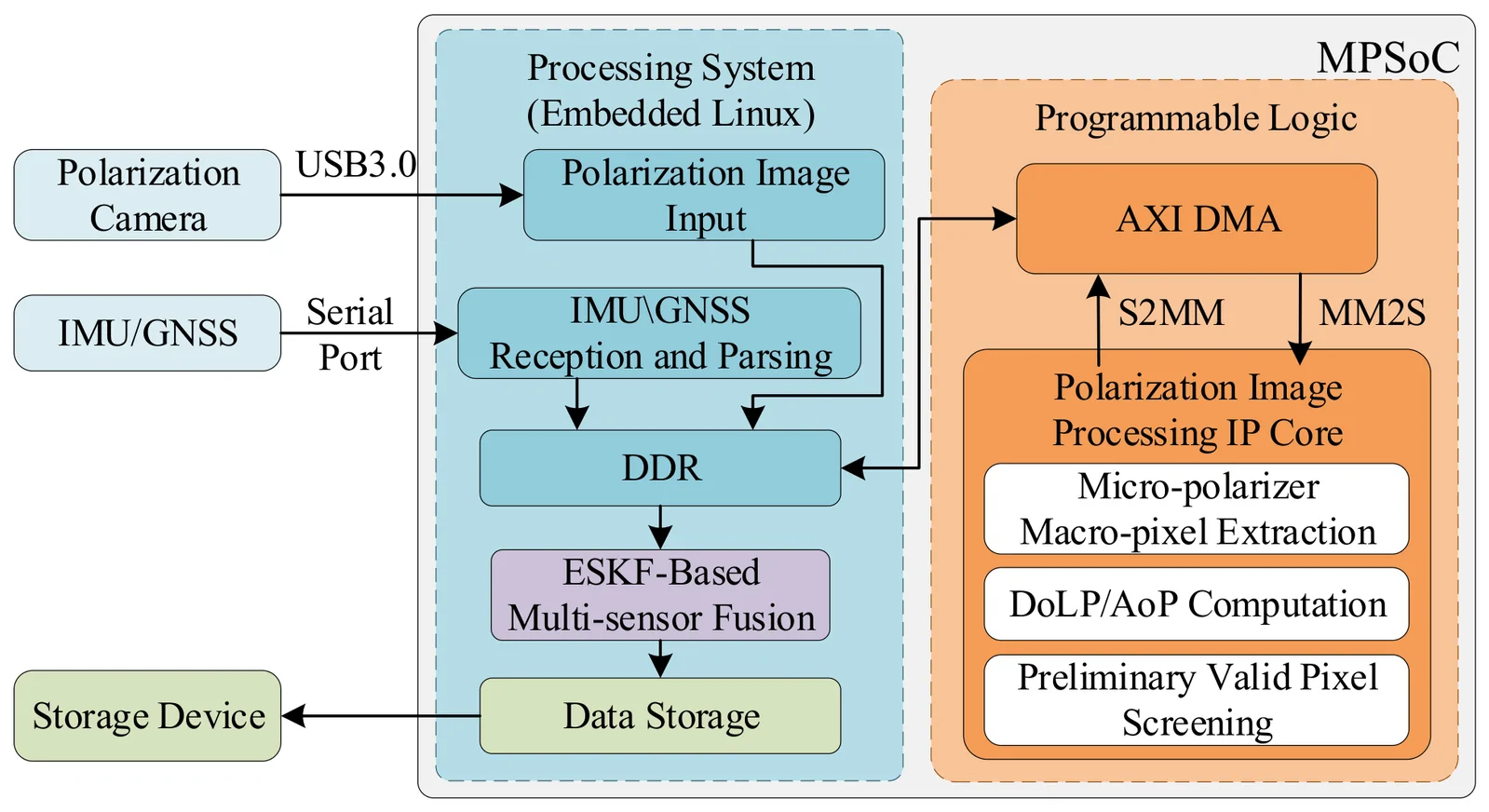
Overall architecture of the proposed system.

**Figure 2:**
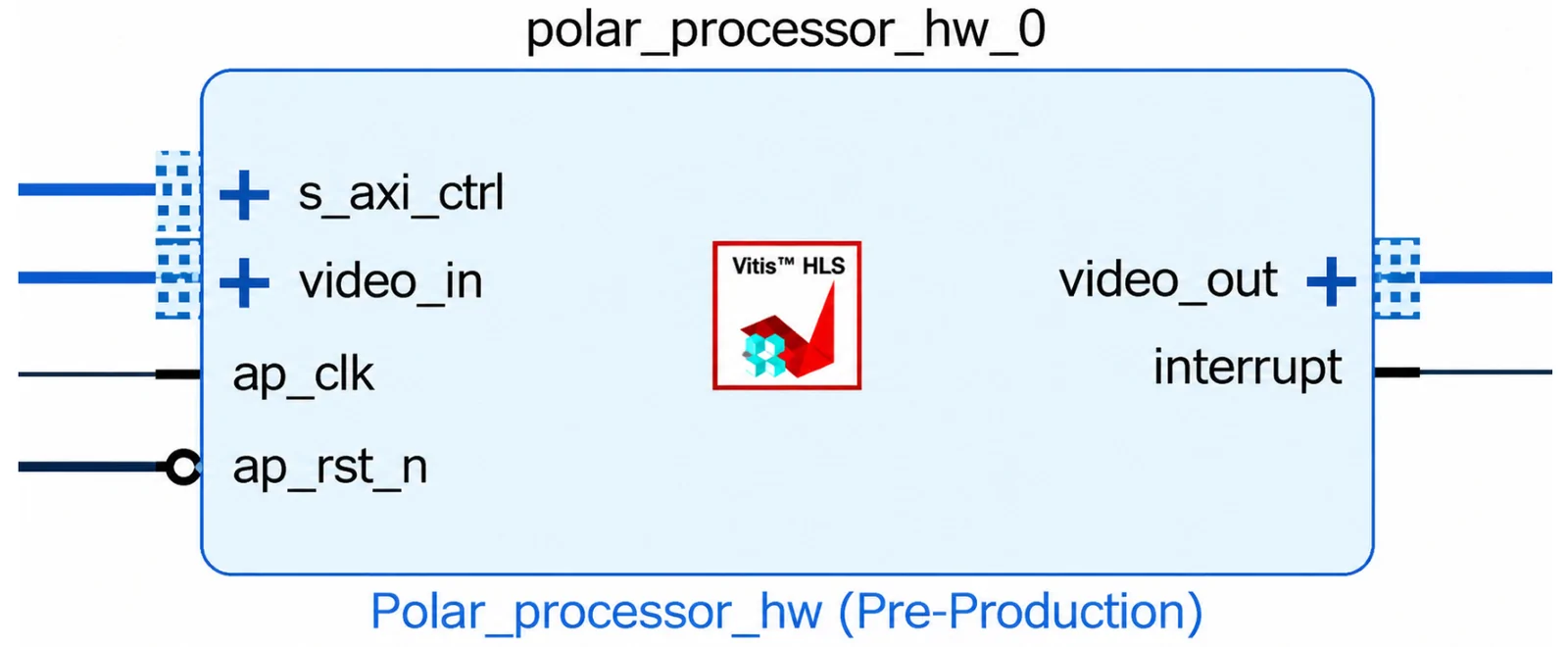
Polarization-image-processing IP core.

**Figure 3:**
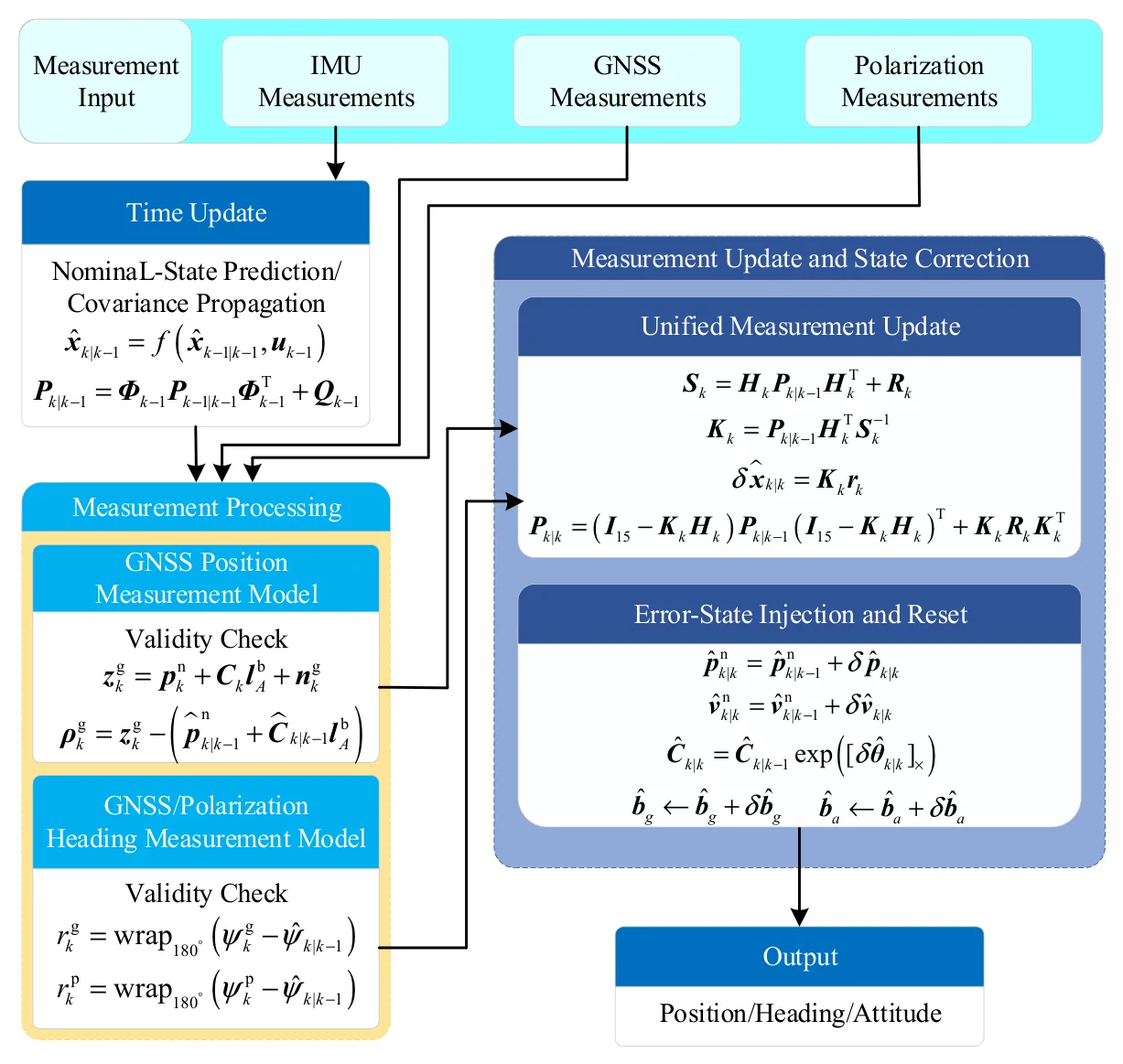
Flowchart of the ESKF-based multi-source fusion algorithm.

**Figure 4:**
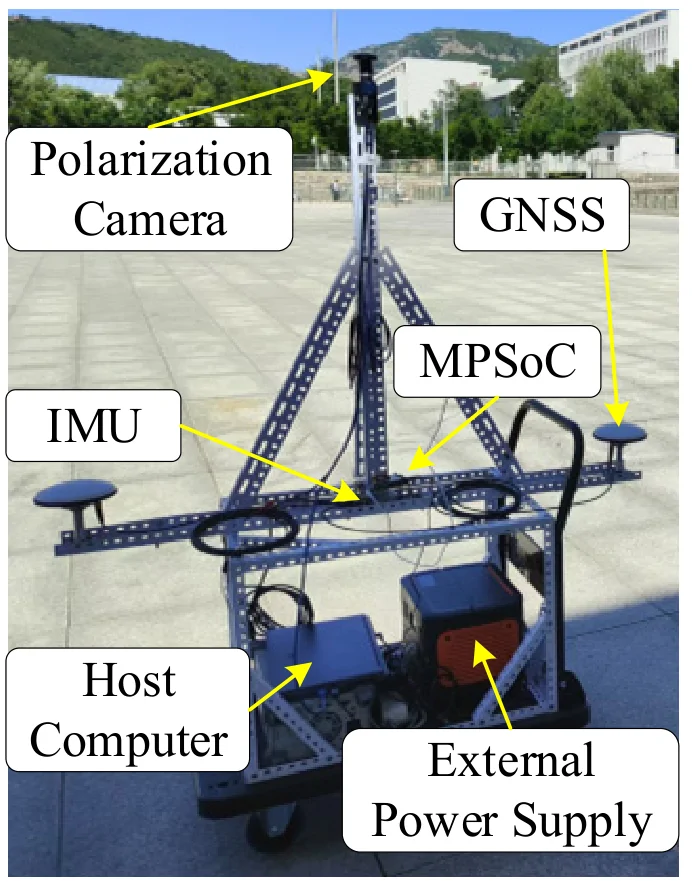
Experimental platform.

**Figure 5:**
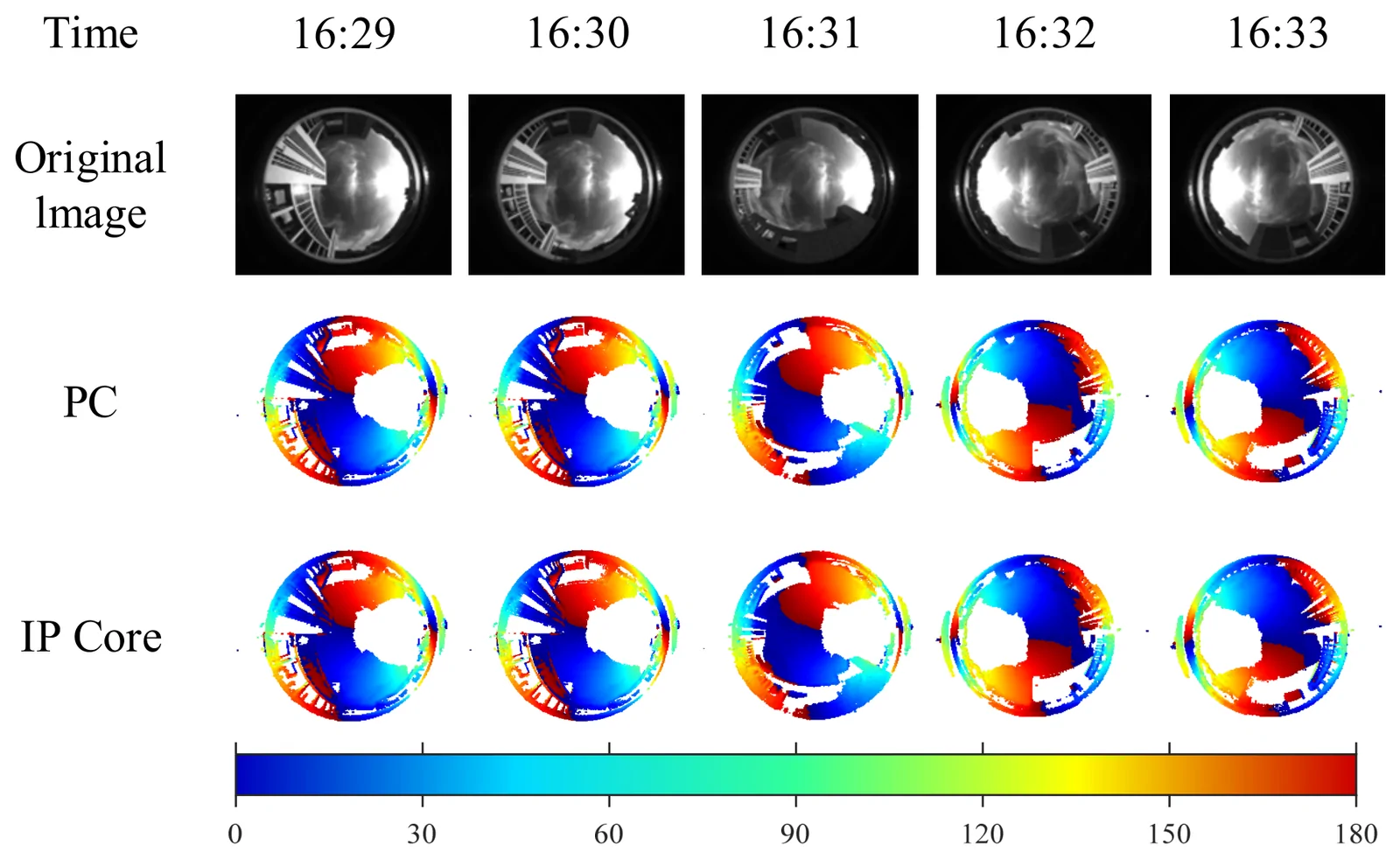
Comparison of AoP calculation results between the PC and the IP core.

**Figure 6:**
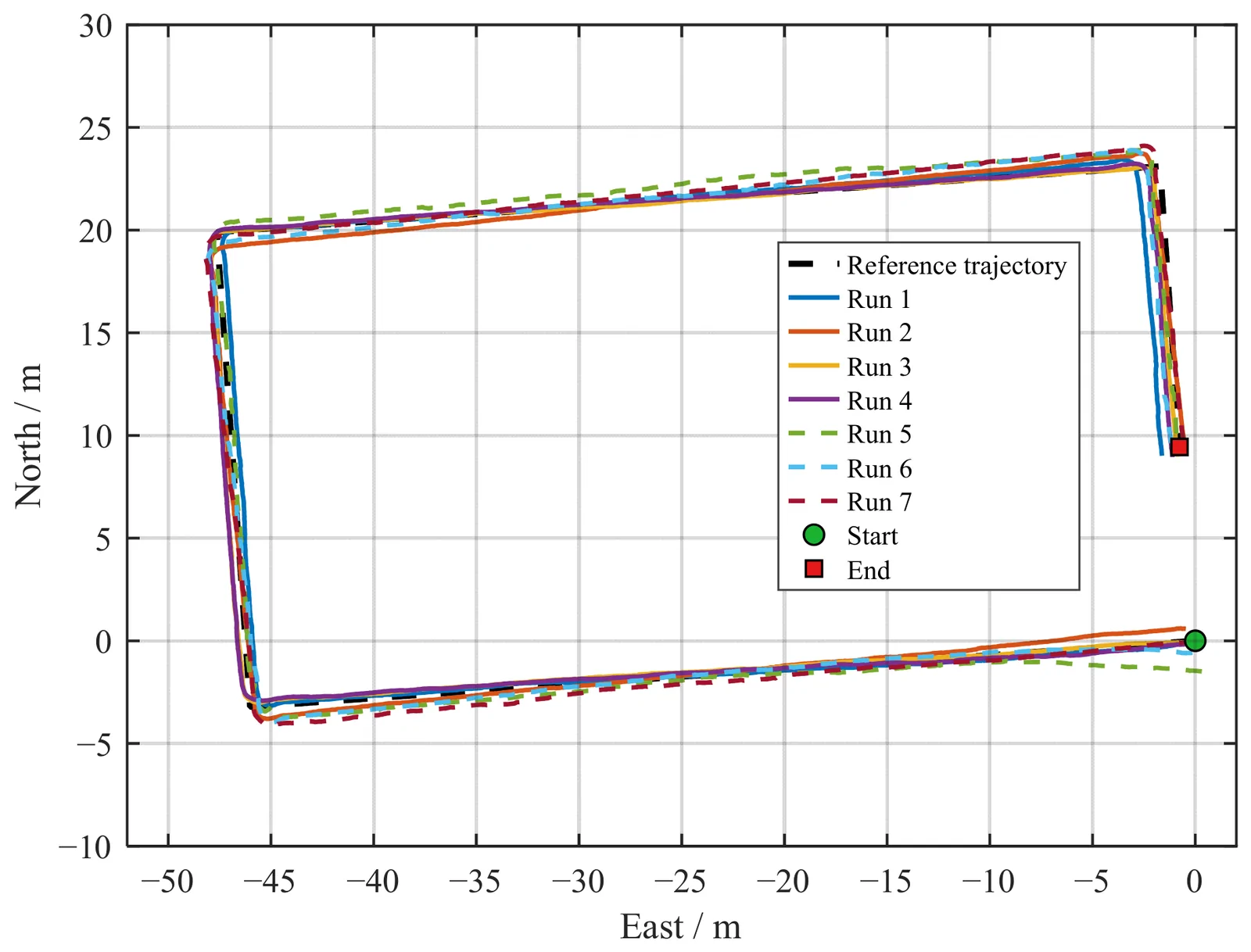
Comparison of trajectories from the seven repeated experiments.

**Figure 7:**
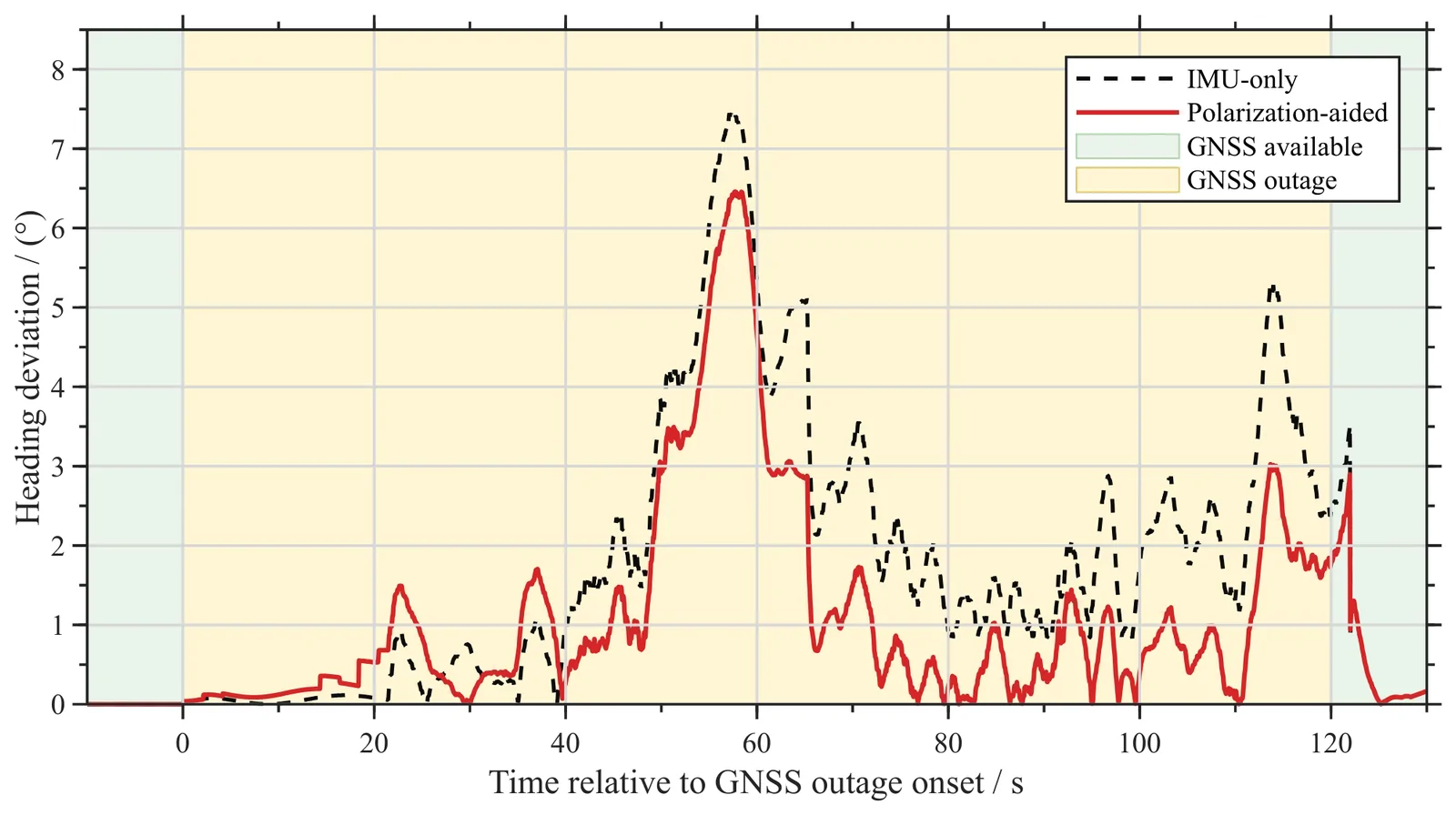
Comparison of heading deviations under GNSS outage.

**Table 1:** HLS results of the polarization-image-processing IP core.

Metric	Result
Target clock period	10.00 ns
Estimated clock period	7.271 ns
LUT	2182
FF	1263
BRAM	7
DSP	5

**Table 2:** Sensor specifications.

Device	Performance Metric	Value
IMU\GNSS	Accelerometer Bias Accelerometer Random Walk Gyroscope Bias Gyroscope Random Walk Data Update Rate	30 μg 0.04 m/s√h 2.5°/h 0.3°/√h 20 Hz
Polarization Camera	Resolution	2448 (H) × 2048 (V)

**Table 3:** Key fusion and calibration parameters.

Parameter	Value
GNSS-derived course construction	time baseline: 2.0 s minimum displacement: 0.35 m freshness threshold: 0.30 s
Minimum vehicle speed	GNSS-derived course: 0.50 m/s polarization observation: 0.12 m/s
Polarization-quality thresholds	DoLP ≥0.10 valid-sky proportion ≥0.03
Polarization-innovation threshold	$10^{\circ }/20^{\circ }$
Polarization-observation standard-deviation range	$12^{\circ }\sim 25^{\circ }$
Installation and calibration parameters	$b_{p}=4.987^{\circ }$; $l_{A}^{b}=[0,\;\;0.525,\;\;0{]}^{T}$ m

**Table 4:** AoP calculation differences.

Run	MAE/°	RMSE/°	Max Error/°
Run 1	0.304	0.367	0.897
Run 2	0.300	0.363	0.892
Run 3	0.303	0.366	0.892
Run 4	0.313	0.375	0.897
Run 5	0.277	0.338	0.892
Run 6	0.295	0.357	0.887
Run 7	0.288	0.350	0.892

**Table 5:** Measured processing performance of the PS-only and PS–PL implementations.

Implementation	Main Pixel-Level Processing Stage (ms)	Frame-Level Statistics (ms)	End-to-End Time (ms)	Sustained Processing Rate (fps)
PS-only	94.976	12.441	107.416	9.310
PS–PL	50.171	11.318	97.163	10.292

**Table 6:** Comparison with representative real-time polarization-image hardware implementations.

Work	Hardware Platform	Input Resolution	Reported Processing Rate	Input-Pixel Throughput (MPixel/s)
York et al. [24]	Embedded FPGA	1000 × 1000	50 fps	50.00
Lapray et al. [25]	Zynq-7000	648 × 488	60.2 fps	19.05
Lu et al. [15]	Zynq-7000	1280 × 1024	10 fps	13.11
This work	Zynq UltraScale+ MPSoC	2448 × 2048	10.292 fps	51.60

**Table 7:** Position deviations relative to the preset trajectory.

Run	MAE/m	RMSE/m	Max Error/m
Run 1	0.336	0.397	0.759
Run 2	0.286	0.452	1.052
Run 3	0.277	0.380	0.757
Run 4	0.279	0.426	0.968
Run 5	1.475	1.784	2.718
Run 6	1.151	1.235	1.669
Run 7	0.618	0.717	1.174

**Table 8:** Heading deviations relative to the preset motion direction.

Run	MAE/°	RMSE/°	Max Error/°
Run 1	1.557	1.814	4.300
Run 2	1.566	1.959	6.125
Run 3	2.256	2.552	5.620
Run 4	1.929	2.351	6.475
Run 5	3.909	4.693	11.082
Run 6	1.803	2.272	6.823
Run 7	3.533	3.775	8.203

**Table 9:** Heading deviations for different GNSS-outage durations.

GNSS Outage/s	IMU-Only MAE/°	Polarization-Aided MAE/°	IMU-Only RMSE/°	Polarization-Aided RMSE/°
20	0.458	0.654	0.537	0.766
30	0.842	0.934	1.045	1.166
40	1.141	0.983	1.378	1.202
50	1.121	1.192	1.352	1.430
60	1.237	1.384	1.563	1.667
90	1.611	1.514	2.275	1.932
120	2.415	1.982	2.922	2.170

## Data Availability

The data are not publicly available due to confidentiality restrictions. The data may be available from the corresponding author upon reasonable request, subject to the applicable confidentiality requirements.
